# The Psychologically Rich Life Questionnaire in China

**DOI:** 10.1002/pchj.812

**Published:** 2024-11-17

**Authors:** Xiang Zhao, Gareth Davey, Xiangxing Wan

**Affiliations:** ^1^ Research Centre for Languages and Cultures, School of Foreign Languages and Literature Yunnan Normal University Kunming China; ^2^ Health Psychology Unit, Institute of Psychology University of Klagenfurt Klagenfurt am Wörthersee Austria; ^3^ Faculty of Professional Studies University of Bolton England UK

**Keywords:** Chinese adolescents, psychological richness, Psychologically Rich Life Questionnaire, psychometrics

## Abstract

Research on psychological richness in China and in adolescents is limited. We validated the 17‐item Psychologically Rich Life Questionnaire in a sample of 1794 Chinese high school students. Internal consistency was adequate, and a two‐factor structure was found.

Psychological richness characterizes a life filled with interesting, varied and perspective‐changing experiences (Oishi and Westgate 2022), in contrast to happiness that refers to positive affect, and meaning in life that refers to the subjective sense that life experiences are coherent and purposeful. Psychological richness seems essential for the heightened need for fulfillment and resilience in the post‐pandemic‐world (Gu, Tao, and Zheng 2023). However, studies in China are scarce. Recent data from a Chinese college student sample using the Psychologically Rich Life Questionnaire (PRLQ; Gu et al. 2023), the most widely used measure of psychological richness, showed a high internal consistency (*α* = 0.93), in agreement with Oishi and Westgate's (2022) conclusion that its Cronbach's alpha (*α*) ranges from 0.87 to 0.93 across cultures. However, no convergent validation studies of the scale have been done in China. We investigate its convergent validity in a sample of Chinese adolescents, to assess theoretical relatedness to happiness and meaning in life and to examine item structure and response patterns and any gender or social status differences.

A sample of 1794 tenth‐graders in 24 secondary schools (62.4% girls; M_age_ = 16.6 years, SD_age_ = 0.6) in six regions of Yunnan Province (a representative sample of provincial socioeconomic status) answered the 17‐item, 7‐point Likert scale version of the PRLQ (Oishi and Westgate 2022; Table 1). The survey also included the presence and search subscales of the Meaning in Life Questionnaire (MLQ; Steger et al. 2006) with a 7‐point Likert scale, a single‐item question from the PERMA‐Profiler measuring happiness with an 11‐point Likert scale (Butler and Kern 2016), and a ladder diagram measuring social status with 10 levels (Goodman et al. 2001). Gender and age variables were also assessed. Survey items were translated from English to simplified Chinese (see Table S1) and reviewed by three bilingual scholars familiar with linguistic expressions in Yunnan Province. The survey was completed online with mobile phones, and all quantitative items were compulsory (i.e., no missing values). The procedure received ethical approval from the appropriate review board at the Yunnan Normal University, and informed consent was obtained from the participants.

**TABLE 1:** Indices of 17 items from the Psychologically Rich Life Questionnaire (Oishi and Westgate 2022).

Items	*H*	*F*
1. My life has been psychologically rich	0.45	0.75
2. My life has been experientially rich	0.50	0.85
3. My life has been emotionally rich	0.47	0.80
4. I have had a lot of interesting experiences	0.55	0.90
5. I have had a lot of novel experiences	0.54	0.90
6. My life has been full of unique, unusual experiences	0.49	0.85
7. My life consists of rich, intense moments	0.50	0.84
8. My life has been dramatic	0.33	0.64
9. I experience a full range of emotions via first‐hand experiences such as travel and attending concerts	0.43	0.72
10. I have a lot of personal stories to tell others	0.45	0.75
11. On my deathbed, I am likely to say “I had an interesting life”	0.50	0.78
12. On my deathbed, I am likely to say “I have seen and learned a lot”	0.48	0.77
13. My life would make a good novel or movie	0.50	0.79
14. My life has been monotonous	0.09	−0.06
15. I often feel bored with my life	0.12	−0.03
16. My life has been uneventful	0.14	−0.00
17. I can't remember the last time I've done or experienced something new	0.12	−0.01

*Note: H* = coefficient of homogeneity. *F* = Standardized factor loading. Items 14 to 17 are reverse coded. All items were answered on a 7‐Likert scale, from [1] *strongly disagree* to [7] *strongly agree*.

The PRLQ demonstrated high internal consistency on Cronbach's alpha (*α* = 0.91), Guttman's lambda 6 (*G6* = 0.95), and McDonald's omega (*ω* = 0.92). The coefficient of homogeneity (*H*) of the whole scale was only 0.39, where items 14 to 17 were unscalable (*H* < 0.30). The exploratory factor analysis among the 17 items showed a better fit of a two‐factor structure over a one‐factor structure (see Table S2), with items 14 to 17 composing the second factor. Similarly, confirmatory factor analysis resulted in a poor fit when all 17 items were regarded as one factor: RMSEA = 0.19, CFI/TLI = 0.72/0.68, SRMR = 0.15. In contrast, when items were grouped as two factors (i.e., factor 1 by the first 13‐items; factor 2 by the last 4‐items), model fit improved: RMSEA = 0.11, CFI/TLI = 0.91/0.89, SRMR = 0.06 (for path diagrams, see Figure S1).^1^ Boys (M = 73.69) reported a higher sum score of psychological richness than girls (M = 71.99), although the discrepancy is negligible given the small effect size (*t*_(1359.5)_ = −2.04, *p* = 0.041, Cohen's *d* = −0.10). Participants who perceived their family as bearing higher social status reported higher psychological richness scores (*r* = 0.18, *p* < 0.001). Regarding convergent validity analyses, psychological richness was positively associated with presence (*r* = 0.58, *p* < 0.001) and search (*r* = 0.51, *p* < 0.001). Psychological richness was positively associated with perceived happiness (*r* = 0.45, *p* < 0.001).

In conclusion, the PRLQ has robust reliability, although its unidimensionality needs further investigation. The high reliability of the 17‐item PRLQ concurs with Gu et al.'s (2023) use of the 12‐item version. The PRLQ demonstrates good convergent validity as it is moderately associated with measures of happiness and meaning in life. This confirms the distinctness of psychological richness from happiness and meaning in life but also their interrelatedness, in agreement with previous work (Oishi and Westgate 2022). The high internal consistency of the 17‐item PRLQ administered in the Chinese language is consistent with data from the English‐language version. However, in our study, the last four items of the 17‐item PRLQ appear to be a separate factor: the first factor can be characterized as “psychological richness” and the second factor as “life mundaneness,” thereby advancing understanding of the PRLQ. Although an original finding, “life mundaneness” supports Oishi and Westgate's (2022) suggestion that people sometimes seek psychological richness at the expense of comfort, making the PRLQ unique among psychological scales. Future research is needed, then, for composite scores of the PRLQ as the sum score may not well capture the bifactorial meaning we have identified. The inconclusive associations between the PRLQ and demographic variables based on effect sizes call for research in the general population and with wider age ranges, although the positive associations between psychological richness and social status in this study concur with reports that happiness tends to be higher for individuals with higher socioeconomic status.

## Conflicts of Interest

The authors declare no conflicts of interest.

## Supporting information


**Data S1** Supporting Information.

## Endnotes

^1^
Following the model modifications by Oishi et al. (2019; sect. 3.1), greater model fit indices were observed when those error terms were associated, RMSEA = 0.08, CFI/TLI = 0.95/0.94, SRMR = 0.05. However, in recognition of methodological criticisms of allowing correlated residuals (Hermida 2015), we maintain the two‐factor model.
