# Supplementary material for: The Psychologically Rich Life Questionnaire in China
Source: Psych J. 2024 Nov 17;14(1):153–4. doi: 10.1002/pchj.812 (PMC11787873; doi:10.1002/pchj.812)
Supplement: Supplementary file 1 — Data S1 Supporting Information. [file PCHJ-14-153-s001.docx]

Supplementary Materials

Table S1

*Simplified Chinese translation of the items in the 17-item Psychologically Rich Life Questionnaire*

| Item |
| --- |
| 1.我的生活在心理上是丰富的。 |
| 2.我的生活经历丰富。 |
| 3.我的生活情感丰富。 |
| 4.我有很多有趣的经历。 |
| 5.我有很多新奇的经历。 |
| 6.我的生活充满了独特、不寻常的经历。 |
| 7.我的生活充满了丰富而紧张的时刻。 |
| 8.我的人生充满戏剧性。 |
| 9.我通过旅行和参加音乐会等亲身经历体验全方位的情感。 |
| 10.我有很多个人故事可以告诉别人。 |
| 11.临终前，我可能会说 “我的生活很有趣”。 |
| 12.临终前，我可能会说“我看到了很多，学到了很多”。 |
| 13.我的生活会成为一部好看的小说或电影。 |
| 14.我的生活很单调。 |
| 15.我经常对自己的生活感到无聊。 |
| 16.我的生活平淡无奇。 |
| 17.我不记得上次做或经历新事物是什么时候。 |

Table S2

*Fit statistics of exploratory factor analysis with one- and two-factorial models*

|  | AIC | BIC | SABIC | Chi-square | df | *p* | CFI | RMSEA |
| --- | --- | --- | --- | --- | --- | --- | --- | --- |
| One-factor | 93761.62 | 93948.35 | 93840.34 | 7566.92 | 119 | <.001 | 0.72 | 0.19 |
| Two-factor | 88400.38 | 88674.99 | 88516.14 | 2173.68 | 103 | <.001 | 0.92 | 0.11 |

Note. Rotation method: Geomin oblique.

Figure S1

*Path diagram of the confirmatory factor analysis for the 17 items in the Psychologically Rich Life Questionnaire*


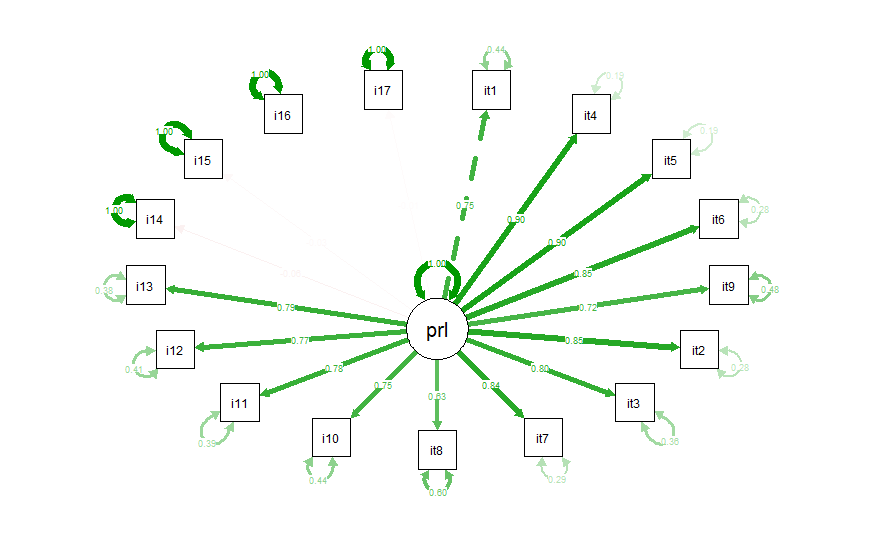


(A) One-factor model


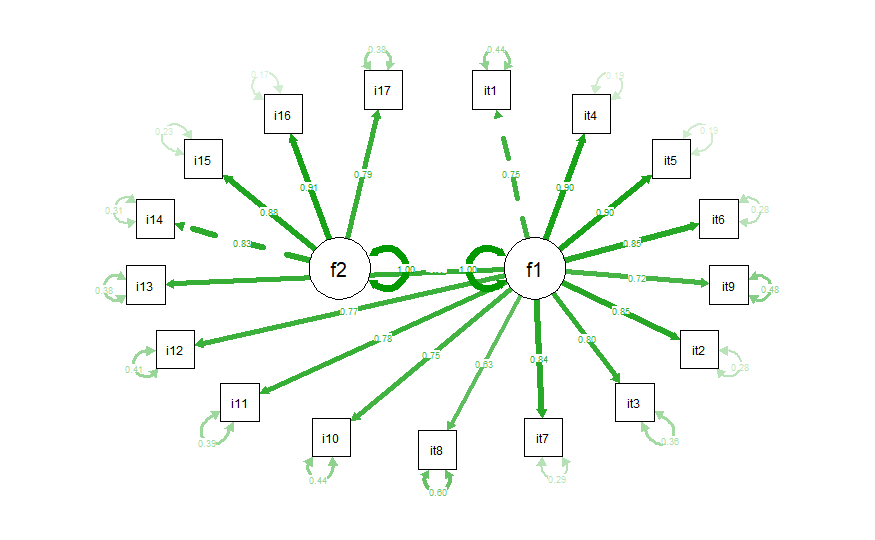


(B) Two-factor model
